# 

**Published:** 1893-12

**Authors:** 


### BOOKS RECEIVED.

A Text-Book of Physiological Chemistry. By Olof Hammarsten, Professor of Medical and Physiological Chemistry in the University of Upsala. Authorized translation from the second Swedish edition, and from the author's enlarged and revised German edition. By John A. Mandel, Assistant to the Chair of Chemistry, etc., in the Bellevue Hospital Medical College, and in the College of the City of New York. First edition ; first thousand. Octavo ; cloth, pp. x.—511, $4.00 New York : John Wiley & Sons, 53 East Tenth Street. 1893.

A Manual for Boards of Health and Health Officers. By Lewis Balch, M. D., Ph. D., Secretary State Board of Health of New York ; Health Officer of Albany ; Emeritus Professor of Anatomy and Professor of Medical Jurisprudence Albany Medical College. Pp. 242. Albany, N. Y.: Banks & Bros. 1893.

The Principles and Practice of Surgery. By John Ashhurst, Jr., M. D., Barton Professor of Surgery and Clinical Surgery in the University of Pennsylvania ; Surgeon to the Pennsylvania Hospital, Philadelphia. New (sixth) edition, enlarged and thoroughly revised. In one octavo volume of 1,161 pages, with 656 engravings and a colored plate. Cloth, $6.00 ; leather, $7.00. Philadelphia : Lea Bros. & Co. 1893.

Duane's Students' Dictionary of Medicine. The Students' Dictionary of Medicine and the Allied Sciences. Comprising the pronunciation, derivation and full explanation of medical terms, together with much collateral descriptive matter, numerous tables, etc. By Alexander Duane, M. D., Assistant Surgeon to the New York Ophthalmic and Aural Institute ; Reviser of Medical Terms for Webster's International Dictionary. In one square octavo volume of 658 pages. Cloth, $4.25 ; half leather, $4.50 ; full sheep, $5.00. Philadelphia : Lea Bros. & Co. 1893.

Chemistry and Physics. By Joseph Struthers, Ph. B., Columbia College School of Mines, New York ; D. W. Ward, Ph. B., Columbia College School of Mines, New York, and Charles H. Willmarth, M. S., New York. $1.00. The Students' Quiz Series. Philadelphia: Lea Bros. & Co. 1893.

A Practical Treatise on Diseases of the Skin. For the use of students and practitioners. By J. Nevins Hyde, A. M., M. D., Professor of Dermatology and Venereal Diseases in Rush Medical College, Chicago. New (third) edition. In one octavo volume of 802 pages, with nine plates, of which three are colored, and 108 engravings. Cloth, $5.00 ; leather, $6.00. Philadelphia : Lea Bros. & Co. 1893.

A System of Genito-Urinary Diseases, Syphilology and Dermatology, by various authors. Edited by Prince A. Morrow, A. M., M. D., Clinical Professor of Genito-Urinary Diseases ; formerly Lecturer on Dermatology in the University of the City of New York ; Surgeon to the Charity Hospital, etc. With illustrations. In three large 8vo volumes. Volume II., Syphilology. Pp. xviii.—917. Sold only by subscription.

Transactions of the American Surgical Association. Volume XI. Edited by DeForest Willard, M. D., Recorder of the Association. Philadelphia : Printed for the Association, and for sale by William J. Dornan. 1893.

International Clinics. A Quarterly of Clinical Lectures on Medicine, Neurology, Pediatrics, Surgery, Genito-Urinary Surgery, Gynecology, Ophthalmology, Laryngology, Otology, and Dermatology. By professors and lecturers in the leading medical colleges of the United States, Great Britain, and Canada. Edited by John M. Keating, M. D., LL.D., Colorado Springs, Col.; Fellow of College of Physicians, Philadelphia ; formerly Consulting Physician for Diseases of Women to St. Agnes' Hospital ; Gynecologist to St. Joseph's Hospital ; Visiting Obstetrician to the Philadelphia Hospital, and Lecturer on Diseases of Women and Children, Philadelphia ; Editor Cyclopedia of the Diseases of Children. Judson Daland, M. D., Philadelphia, Instructor in Clinical Medicine, and Lecturer on Physical Diagnosis and Symptomatology in the University of Pennsylvania ; Assistant Physician to the University Hospital ; Physician to the Philadelphia Hospital and to the Rush Hospital for Consumption. J. Mitchell Bruce, M. D., F. R. C. P., London, England, Physician and Lecturer on Therapeutics at the Charing Cross Hospital. David W. Finlay, M. D., F. R. C. P., Aberdeen, Scotland, Professor of Practice of Medicine in the University of Aberdeen ; Physician to, and Lecturer on, Clinical Medicine in the Aberdeen Royal Infirmary ; Consulting Physician to the Royal Hospital for Diseases of the Chest, London. Volume III. Third series. Royal octavo, pp. xii.—356. Philadelphia : J. B. Lippincott Co. 1893.

United States Department of Agriculture. Weather Bureau Current Chart of the Great Lakes. By Mark W. Harrington, Chief of Weather Bureau, Washington, D. C. 1893.

### Literary Notes.

American Text-Book of Gynecology.—Mr. W. B. Saunders, publisher, of Philadelphia, Pa., announces this work as ready for early issue. It is the joint production of Drs. Howard Kelley, Pryor, Byford, Baldy, Tuttle, and others who stand before the profession for all that is progressive in gynecology. The work will contain operations not before described in any other book—notably, ablation of fibroid uterus. It is designed as a profusely illustrated reference book for the practitioner, and every practical detail of treatment is precisely stated.

---

Mark Twain's Latest—Romance of an Esquimau Maiden.—A magazine is usually satisfied with one strong feature for the month. *The Cosmopolitan*, however, presents for November no less than five very unusual ones. William Dean Howells gives the first of the letters of the traveler, who has been visiting this country, from Altruria. We have read Mr. Howell's impression of the Altrurian; but in this first letter we have the Altrurian's impressions of New York, with some comments upon our government and society, calculated to awaken the most conservative minds. The second feature of *The Cosmopolitan* is the portion of the magazine given up to color work, no less than ten superb color illustrations being presented for the first time in magazine history, accompanying an article by Mrs. Roger A. Pryor on Changes in Women's Costumes. The third feature is American Notes, by Walter Besant, who was recently in America, and is doing the United States for *The Cosmopolitan*, *a la* Dickens. The fourth feature is an article by General Badeau on The Forms of Invitation Used by the English Nobility. The article is illustrated by the *fac-simile* of cards to the Queen's drawing-room, to dinner at the Princess of Wales, and to many leading houses of England. Finally, we have a new and very curious story by Mark Twain, called The Esquimau Maiden's Romance. It is in his happiest vein, and is illustrated by Dan Beard. The November number presents the work of many artists, among whom are: C. S. Reinhart, Otto Guillonnet, J. H. Harper, G. Hudson, Franz von Lenbach, George Wharton Edwards, F. Schuyler Matthews, Dan Beard, W. L. Sontag, Jr., F. G. Attwood, C. Hirschberg, J. Habert-Dys, August Franzen, Louis J. Read, J. N. Hutchins, and Hamilton Gibson.

Sousa's new march, The Manhattan Beach March, has been purchased by *The Ladies' Home Journal*, and its full piano score will be printed in the Christmas issue. The composer claims for it a superiority over either his popular Washington Post or High School Cadets marches.

Notice to Contributors.—We are glad to receive contributions from every one who knows anything of interest to the profession. Articles designed for publication in the Journal should be handed in before the first day of the month. The Editors are not responsible for the views or opinions of contributors. All communications should be addressed to the Managing Editor, 284 Franklin St., Buffalo, N. Y.

